# Differences in Innate Immunologic Response to Group B Streptococcus Between Colonized and Noncolonized Women

**DOI:** 10.1155/S1064744901000230

**Published:** 2001

**Authors:** Jennifer M. Smith, Rachel H. Respess, David G. Chaffin, Bryan Larsen, Susan H. Jackman

**Affiliations:** 1 Departments of Microbiology Immunology and Molecular Genetics and Obstetrics and Gynecology Marshall University School of Medicine Huntington WV USA; 2 Department of Obstetrics and Gynecology Marshall University School of Medicine 1542 Spring Valley Drive Huntington WV 25704 USA; 3 Des Moines University Osteopathic Medical Center Des Moines IA USA

## Abstract

*Objective:* To evaluate the functional capacity of granulocytes and monocytes from pregnant and nonpregnant women in relation to group B streptococcus (GBS) colonization status.

*Methods: *Engulfment of fluorescent GBS by peripheral blood phagocytes from GBS-colonized and noncolonized women was measured by flow cytometry. Intracellular superoxiode generated in response to GBS challenge to monocytes and granulocytes enriched from peripheral blood of these women was also measured by flow cytometry, and extracellular superoxide was determined by colorimetric assay.

*Results:* Monocytes and granulocytes from pregnant, GBS-colonized women engulfed significantly greater numbers of GBS than phagocytes from pregnant, noncolonized women. No difference in intracellular superoxide production was detected between any of the groups of women; however, monocytes from pregnant, colonized women released significantly more superoxide into the extracellular milieu than did granulocytes from the same women. No differences in extracellular release of superoxide were observed among noncolonized women whether they were pregnant or not.

*Conclusions:* Monocytes from pregnant, colonized women engulf more GBS and release more of the superoxide into the extracellular environment, where it is unlikely to be an effective defense mechanism against intracellular bacteria. This suggests that components of the innate immune system that should serve in a protective role may function suboptimally, thereby contributing to the colonization process by GBS.


*Infect Dis Obstet Gynecol 2001;9:125–132*

### Differences in innate immunologic response to group B streptococcus between colonized and noncolonized women

**Jennifer M. Smith[1], Rachel H. Respess[2], David G. Chaffin[2], Bryan Larsen[3] and Susan H. Jackman[1]**

[1]*Departments of Microbiology, Immunology and Molecular Genetics and Obstetrics and Gynecology, Marshall University School of Medicine, Huntington, WV*
[2]*Department of Obstetrics and Gynecology, Marshall University School of Medicine, Huntington, WV*
[3]*Des Moines University Osteopathic Medical Center, Des Moines, IA*

***Objective:*** To evaluate the functional capacity of granulocytes and monocytes from pregnant and nonpregnant women in relation to group B streptococcus (GBS) colonization status.
***Methods:*** Engulfment of fluorescent GBS by peripheral blood phagocytes from GBS-colonized and noncolonized women was measured by flow cytometry. Intracellular superoxiode generated in response to GBS challenge to monocytes and granulocytes enriched from peripheral blood of these women was also measured by flow cytometry, and extracellular superoxide was determined by colorimetric assay.
***Results:*** Monocytes and granulocytes from pregnant, GBS-colonized women engulfed significantly greater numbers of GBS than phagocytes from pregnant, noncolonized women. No difference in intracellular superoxide production was detected between any of the groups of women; however, monocytes from pregnant, colonized women released significantly more superoxide into the extracellular milieu than did granulocytes from the same women. No differences in extracellular release of superoxide were observed among noncolonized women whether they were pregnant or not.
***Conclusions:*** Monocytes from pregnant, colonized women engulf more GBS and release more of the superoxide into the extracellular environment, where it is unlikely to be an effective defense mechanism against intracellular bacteria. This suggests that components of the innate immune system that should serve in a protective role may function suboptimally, thereby contributing to the colonization process by GBS.

Key words: PHAGOCYTOSIS; SUPEROXIDE; FLOW CYTOMETRY

Over the past 30 years group B streptococcus (GBS) has gained prominence as the primary etiologic agent of sepsis, bacteremia and meningitis in the neonatal period. Administration of intrapartum antibiotics to women identified at risk for transmitting GBS can be effective in prevention of transmission and early-onset disease[1], but it fails to address the underlying problem of maternal colonization. Despite estimates that between 15 and 35% of women are asymptomatically colonized with GBS[2], little is known as to why GBS can evade the normal immune response in some women (colonized) and not in others (noncolonized).

Phagocytic cells, particularly monocytes and granulocytes, are key mediators of innate

Correspondence to: Susan H. Jackman, PhD, Department of Microbiology, Immunology and Molecular Genetics, Marshall University School of Medicine, 1542 Spring Valley Drive, Huntington, WV 25704, USA. Email: jackman@marshall.edu immunity to microorganisms, providing an early, rapid response to a potential pathogen while the subsequent antigen-specific response is being initiated. Phagocytosis of an organism stimulates production of microbicidal reactive oxygen species, including superoxide radicals and hydrogen peroxide. Microbial degradation products generated from the combined effects of the respiratory burst and cytolytic enzymes are then processed and presented to induce either a T cell- or an antibody-mediated response. Alterations of these early steps in defense can lead to ineffective clearance of the microorganism. While extreme examples of these defects, such as leukocyte adhesion deficiencies[3] or respiratory burst defects (chronic granulomatous disease)[4], result in severe, chronic bacterial and fungal infections, more subtle changes due to the unique physiologic adaptations of pregnancy may be involved in the infectious conditions that are particular threats to mothers and their newborns.

To evaluate the role that alterations in early innate immune responses to GBS may play in supporting the colonization process, the phagocytic and respiratory burst capacities of GBS-colonized and -noncolonized women were compared. Because GBS colonization is primarily an obstetric concern and the hormonal changes of pregnancy influence immunologic functioning, comparisons were also made between pregnant and nonpregnant women irrespective of colonization status.

In this study we show that while pregnant women display an increased engulfment of GBS, superoxide release from the phagocytic cells may be increased, providing a possible explanation for colonization and modified adaptive immune response in pregnancy.

### SUBJECTS AND METHODS

### Study population

The populations studied consisted of pregnant and nonpregnant women recruited from Marshall University School of Medicine and its associated obstetric clinics. All patients were at least 18 years of age, had no other known active infections or immunodeficiency, and were not taking antibiotics in the previous 6 weeks. The study was approved by the Marshall University School of Medicine Institutional Review Board, and each woman gave informed consent prior to participation. Peripheral blood from each participant was drawn from the antecubital vein into EDTA Vacutainer tubes (Becton Dickinson, Franklin Lakes, NJ). Amies clear StarSwabs (Starplex Scientific, Ontario, Canada) were used to collect specimens for determination of GBS colonization status. Swabs of the lower third of the vagina from pregnant patients were sent to the Cabell Huntington Hospital laboratory as part of routine obstetric screening. Self-collected swabs from the nonpregnant patients were taken to the research laboratory. All swabs were incubated in Lim broth for 12 h at 37°C in 5% $CO_2$. Aliquots of the Lim broth culture were streaked onto tryptic soy agar with 5% sheep blood, and incubated overnight at 37°C in 5% $CO_2$. Presumptive identification of GBS was based on colony morphology, hippurate catalysis and CAMP test (research laboratory only). Colonies were confirmed as group B streptococcus by latex agglutination (Meritec Strep Group B Beta-hemolytic Streptococcus grouping set; Meridian Diagnostics, Inc., Cincinnati, OH) by both laboratories.

Based on pregnancy status and the results from the vaginal swab cultures the women were placed into four groups: pregnant, GBS-colonized ($n$ = 9 for phagocytic assays; $n$ = 5 for superoxide assays); pregnant, noncolonized ($n$ = 8 for phagocytic assays; $n$ = 5 for superoxide assays); nonpregnant, GBS-colonized ($n$ = 3 for all assays); and nonpregnant, noncolonized ($n$ = 3 for all assays). For the pregnant, GBS-colonized women the mean age was 26.5 years, gravidity ranged from 1 to 7 and parity ranged from 0 to 4. For the pregnant, noncolonized women the mean age was 23.1 years, gravidity ranged from 1 to 6 and parity ranged from 0 to 2. For the nonpregnant, GBS-colonized women the mean age was 38.7 years and gravidity and parity were both 0. For the nonpregnant, noncolonized women the mean age was 30.3 years and gravidity and parity were both 0. All women were Caucasian with the exception of one pregnant, noncolonized woman who was of American Indian heritage. All women were healthy volunteers with medical insurance, and with an estimated annual family income of greater than $15 000.

### Bacterial preparation

Group B streptococci isolated from asymptomatic, colonized women during routine gynecologic exams were assigned the designations 2118 and 2407, corresponding to randomly assigned patient identification numbers. GBS 2118 and 2407 were serotyped as type V and type III, respectively, using a hemolytic streptococcus group B typing sera kit (Accurate Chemical & Scientific Corp., Westbury, NY). GBS 2118 was used for the phagocytic engulfment assays owing to poor fluorescence labeling of type III isolates, such as GBS 2407 used in the superoxide assays.

For the phagocytic engulfment assays, GBS 2118 was labeled by incubation with fluorescein isothiocyanate (FITC) 1 hydrochloride (Research Organincs, Cleveland, OH) at 50 μg/ml in Todd–Hewitt broth for 16 h at 37°C in 5% $CO_2$. The cultures were centrifuged, washed twice with phosphate-buffered saline (PBS) and heat-killed at 60°C for 45 min prior to resuspension to a final concentration of $10^8$ bacteria/ml. After confirming staining via flow cytometry (FACScan, Becton Dickinson, San Jose, CA) the FITC–GBS were aliquoted and stored at −70°C until needed.

For the superoxide assays, GBS 2407 was inoculated from frozen stocks and cultured in Todd–Hewitt broth at 37°C in 5% $CO_2$ until an optical density at 600 nm ($OD_{600}$) of 0.4 was reached (approximately 4 h).

### Phagocytic engulfment

Plasma was removed from 100 μl of whole blood. The cell pellets were washed twice in Dulbecco's phosphate-buffered saline (DPBS) and resuspended in 100 μl heat-inactivated, pooled, human type AB serum (Sigma, St Louis, MO). The pooled human type AB serum failed to agglutinate GBS 2118 in a slide agglutination test. FITC–GBS 2118 was sonicated briefly to create a single-cell suspension prior to adding 10 μl to each tube of resuspended leukocytes. After incubation at 37°C on a rotary shaker for the appropriate length of time, cytochalasin D (Sigma, St Louis, MO) was added to each tube (5 μmol/l final concentration). The contents of each tube were diluted with ice-cold DPBS, centrifuged and washed twice with cold DPBS. Erythrocytes were lysed using ImmunoLyse (Coulter, Miami, FL) according to the manufacturer's instructions. The leukocyte pellet was washed twice with DPBS and the cells were stained for CD14 (phycoerythrin-labeled clone TUK1; Caltag Laboratories, Burlingame, CA) to allow for differentiation of leukocyte populations using flow cytometry. Stained cells were fixed in 4% paraformaldehyde and analyzed by flow cytometry. Side light scatter properties and CD14 staining were used to discriminate between monocytes and granulocytes. A minimum of 5000 events was collected for each population, and analyzed for the percentage of cells having engulfed FITC–GBS and for mean fluorescence intensity using CellQuest Software (Becton Dickinson).

### Superoxide assays

Enriched populations of monocytes and granulocytes were used for measurements of superoxide generated subsequent to engulfment of GBS 2407. Monocytes were enriched from whole peripheral blood by density gradient centrifugation using Ficoll Paque Plus (Amersham Pharmacia Biotech, Piscataway, NJ), followed by negative selection using the StemSep human monocyte enrichment system (Stem Cell Technologies, Vancouver, BC Canada). Granulocytes were enriched from the erythrocyte–granulocyte pellet from the density gradient centrifugation by dextran sedimentation (500 000 average molecular weight; Sigma), followed by hypotonic saline erythrocyte lysis. Viability was assessed by trypan blue staining, and a minimum viability of 90% was required for use in any assay. Purity of the enriched monocyte populations was demonstrated using flow cytometry. Pure monocytes were positive for CD14 and negative for CD3 (clone UCHT1; BD PharMingen, San Diego, CA) and CD19 (clone HIB19; BD PharMingen). Purity of the enriched granulocyte populations was demonstrated using forward and side light scatter properties via flow cytometry.

One hundred-microliter aliquots of enriched monocytes or granulocytes ($1 \times 10^6$ cells/ml) in

PBS-gel were loaded with hydroethidine (10 μmol/l final concentration; Polysciences Inc., Warrington, PA) for 15 min at 37°C. The cells were stimulated by the addition of either phorbol 12-myristate 13-acetate (PMA) at a 200 ng/ml final concentration or 10 μl of log-phase GBS 2407. The fluorescence level was immediately determined by flow cytometry and was used as the zero time point. The granulocytes and monocytes were incubated for 20 min and 45 min, respectively, at 37°C with the stimulators before the final fluorescence reading was taken. A minimum of 5000 events were collected for each population, and analyzed for the percentage of cells with an increase in fluorescence over time after stimulation.

In order to measure superoxide released from the monocytes and granulocytes, 100 μl aliquots of cells ($1 \times 10^6$/ml) in PBS-gel were incubated for 45 min at 37°C in 5% $CO_2$ with 25 μl cytochrome c (2.7 mg/ml; from horse heart), in the presence (25 μl) or absence of superoxide dismutase (1 mg/ml; SOD; from bovine erythrocytes), and with either 200 ng/ml PMA or 10 μl of log-phase GBS 2407. Monocyte- and granulocyte-free tubes were prepared as controls. After incubation the tubes were centrifuged and the supernatants were transferred to a 96-well plate. The absorbance at 550 nm was read and used to calculate the nanomoles of superoxide released according to the following formula: (experimental – matched cell-free control) – (experimental with SOD – matched cell-free control)/47.4.

### Statistical analysis

Statistical analyses were performed using the SigmaStat software package (Jandel Scientific Software, San Rafael, CA). Student's *t*-test was used for comparisons of pregnant versus nonpregnant women and colonized versus noncolonized women. A value of $p < 0.05$ was considered statistically significant. The *t*-test sample size program ($\alpha = 0.05$ and power = 0.8) was used to estimate the number of individuals needed to provide statistical significance when $p > 0.05$.

### RESULTS

### Phagocytic engulfment

The percentages of monocytes and granulocytes having engulfed FITC–GBS 2118 were measured by flow cytometry and compared between GBS-colonized and -noncolonized women. No significant differences were observed between these groups of women regardless of pregnancy status (Table 1).

Although no differences in the percentages of phagocytes having engulfed FITC–GBS 2118 were observed, measurements of mean fluorescence intensity revealed a significant difference in the relative number of FITC–GBS 2118 taken up by phagocytes from pregnant, colonized women when compared with pregnant, noncolonized women. Granulocytes from pregnant, colonized women engulfed approximately twice the number of FITC–GBS 2118 after 20 and 45 min compared

**Table 1** Percentages (mean ± SD) of phagocytes having engulfed fluorescein isothiocyanate–group B streptococcus (FITC–GBS) 2118 over time

| | | | *% Phagocytes containing GBS* | |
|---|---|---|---|---|
| *Pregnancy status* | *Colonization status* | *Cell type* | *20 min* | *45 min* |
| Pregnant | Colonized | Monocytes | 55.9 ± 7.8 | 69.1 ± 12.8 |
| | (*n* = 9) | Granulocytes | 58.6 ± 18.6 | 79.1 ± 14.3 |
| | Noncolonized | Monocytes | 34.7 ± 7.8 | 42.0 ± 19.2 |
| | (*n* = 8) | Granulocytes | 29.4 ± 11.4 | 40.7 ± 18.7 |
| Nonpregnant | Colonized | Monocytes | 48.4 ± 14.9 | 55.5 ± 14.1 |
| | (*n* = 3) | Granulocytes | 52.3 ± 21.2 | 67.3 ± 19.4 |
| | Noncolonized | Monocytes | 37.4 ± 15.5 | 44.1 ± 11.6 |
| | (*n* = 3) | Granulocytes | 25.6 ± 9.8 | 37.1 ± 30.0 |

with those from pregnant, noncolonized women (Figure 1a). Monocytes from pregnant, colonized women engulfed more than five times the number of FITC–GBS 2118 after 45 min compared with those from pregnant, noncolonized women (Figure 1a). These differences were not observed for nonpregnant women (Figure 1b) or in parallel experiments substituting *Staphylococcus aureus* (ATCC 25923) for GBS 2118 (data not shown).

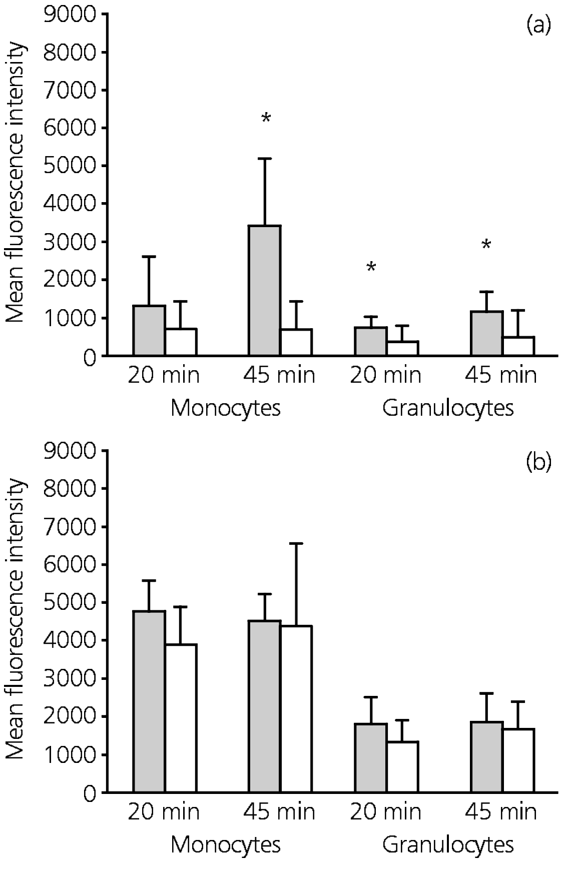

**Figure 1** Mean fluorescence intensity of engulfed fluorescein isothiocyanate–group B streptococcus (FITC–GBS) 2118 over time. Monocytes and granulocytes from colonized (black bars) and noncolonized (white bars), pregnant (a) and nonpregnant (b) women were incubated with FITC–GBS 2118 for 20 or 45 min. Mean fluorescence intensity was measured using flow cytometry. Significant differences between colonized and noncolonized women are denoted by asterisks ($p < 0.05$ using Student's $t$-test). Pregnant, colonized: $n = 9$; pregnant, noncolonized: $n = 8$; nonpregnant, colonized: $n = 3$; nonpregnant, noncolonized; $n = 3$

### Superoxide assays

Preliminary experiments were conducted to determine the time and PMA concentration required for optimal superoxide production. The concentrations and time points used in both superoxide assays reflected maximal PMA as well as GBS stimulation.

A comparison of the percentages of enriched monocytes and granulocytes producing intracellular

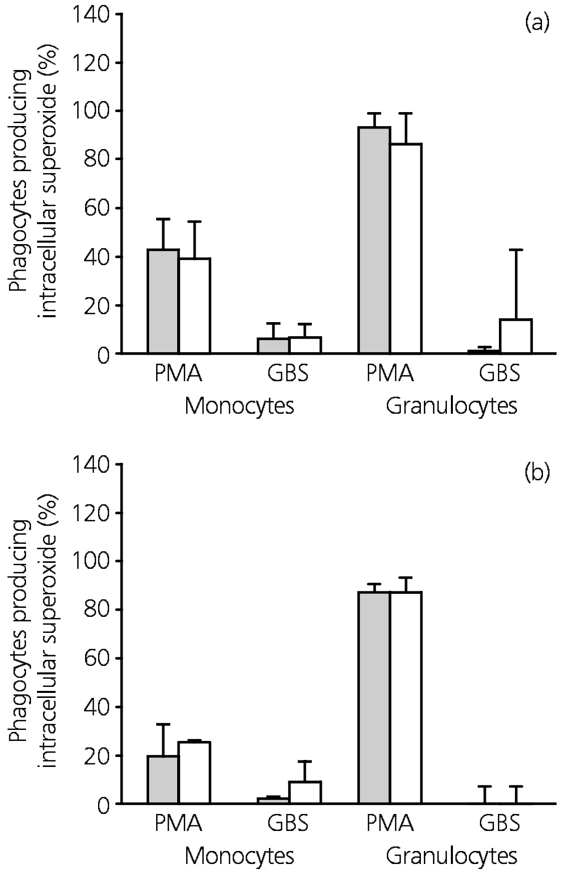

**Figure 2** Generation of intracellular superoxide after stimulation with phorbol 12-myristate 13-acetate (PMA) or group B streptococcus (GBS) 2407. Enriched populations of monocytes and granulocytes from colonized (black bars) and noncolonized (white bars), pregnant (a) and nonpregnant (b) women were loaded with hydroethidine prior to incubation at 37°C with PMA (200 ng/ml) or log-phase GBS 2407. The increase in fluorescence due to superoxide oxidation of hydroethidine to ethidium bromide was measured using flow cytometry at 20 min (granulocytes) and 45 min (monocytes) after stimulation. No significant differences among any of the groups were observed. Pregnant, colonized and noncolonized: $n = 5$; nonpregnant, noncolonized: $n = 3$

superoxide in response to challenge with log-phase GBS 2407 failed to show significant differences between GBS-colonized and noncolonized women, regardless of pregnancy status (Figure 2).

Not all superoxide generated intracellularly remains as such. Extracellular release of superoxide by enriched monocytes and granulocytes in response to challenge with log-phase GBS 2407 was measured spectrophotometrically after 45 min. No significant differences between colonized and noncolonized women were detected. However, monocytes from pregnant, colonized women released a significantly greater amount of superoxide (0.78 ± 0.8 nmol) into the extracellular environment than did the granulocytes from these same women (0.0 ± 0.0 nmol) (Figure 3a). This difference was not observed for noncolonized women regardless of pregnancy status (Figure 3a and b). Parallel experiments with *S. aureus* 25923 again demonstrated the specificity of these differences in relation to GBS (data not shown).

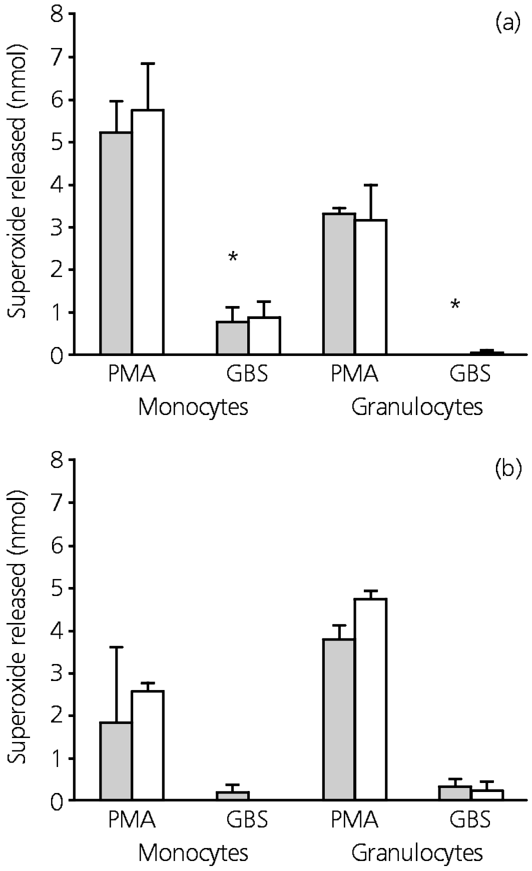

**Figure 3** Release of superoxide into the extracellular milieu following stimulation with phorbol 12-myristate 13-acetate (PMA) or group B streptococcus (GBS) 2407. Enriched populations of monocytes and granulocytes from colonized (black bars) and noncolonized (white bars), pregnant (a) and nonpregnant (b) women were stimulated with PMA (200 ng/ml) or log-phase GBS 2407 for 45 min at 37°C. The nanomoles of superoxide released were calculated from changes in absorbance at 550 nm. Significant differences between monocytes and granulocytes from pregnant, colonized women are denoted by asterisks ($p < 0.05$ using Student's $t$-test). Pregnant, colonized and noncolonized: $n = 5$; nonpregnant, colonized and noncolonized: $n = 3$

### DISCUSSION

Despite recent efforts to devise more accurate and cost-effective methods for identifying and treating pregnant women at risk of transmitting GBS to their newborns, much remains to be learned concerning basic immunity to GBS. Perhaps the most important issue in this area is why certain women become colonized with GBS while others effectively eliminate it. We have proposed that the way in which the innate immune system in GBS-colonized women responds to GBS differs from that in noncolonized women, and that these differences may assist in the initiation of colonization. To test this proposal we compared the abilities of phagocytes from colonized and noncolonized women to engulf GBS and generate the microbicidal superoxide radical following engulfment. Possible contributions owing to pregnancy were also evaluated by comparing responses from pregnant and nonpregnant women.

While no differences in the number of phagocytes having engulfed GBS were observed for any of the groups of women, the bacterial load in phagocytes from pregnant, colonized women was significantly greater than for pregnant, noncolonized women. This effect was probably not due to opsonic antibody, since heterologous serum that failed to agglutinate GBS was used in these assays. Both granulocytes and monocytes from pregnant, GBS-colonized women engulfed greater numbers of GBS. Although overall enhanced phagocytic ability is frequently observed during pregnancy[5–7], the greater than five-fold increase in engulfment by monocytes and the two-fold increase in engulfment by granulocytes from pregnant, GBS-colonized women appears to be specific to GBS, as these differences were not observed in parallel experiments with *S. aureus*.

Following phagocytic engulfment of GBS, superoxide is generated within the phagocyte by phagosome-associated, reduced nicotinamide adenine dinucleotide phosphate (NADPH) oxidase. Not surprisingly, differences with regard to the percentage of phagocytes producing superoxide were not seen, but there was a difference with respect to pregnant, GBS-colonized women in the amount of superoxide released from monocytes versus granulocytes. The release of superoxide into the extracellular environment was greater from monocytes in pregnant, GBS-colonized women. Granulocytes from these women failed to release any superoxide extracellularly.

The mechanism underlying the release of superoxide generated within the phagosome into the extracellular environment is unclear. Two possible explanations can be envisioned, one phagocyte-associated and one GBS-associated. The respiratory burst associated with phagocytic engulfment is activated following specific microbe–phagocyte receptor interactions. If the respiratory burst is activated through these interactions prior to complete closure of the phagosome, superoxide could leak into the extracellular environment. Alternatively, factors produced by GBS may break down the phagocyte membrane integrity, leading to leakage of superoxide outside the cell. Studies have shown that GBS can cross various types of eukaryotic cell membranes, including those from human chorion cells[8], human umbilical vein endothelial cells[9], human lung epithelial cells[10] and human monocytes[11]. Using GBS serotype III strains producing different levels of β-hemolytic activity[9,10], or using agents that inhibit GBS β-hemolysin[11], it has been demonstrated that the β-hemolysin produced by GBS disrupts cellular membranes, alters membrane permeability and causes the release of lactate dehydrogenase. Based on the findings of these studies it can be argued that the β-hemolysin produced by GBS 2407, the serotype III isolate used in our superoxide assays, may damage the plasma and/or phagosome membranes, leading to a release of superoxide into the extracellular environment. Both of these possible mechanisms for the release of superoxide are subjects for future investigations, perhaps by using strains with a deletion of the β-hemolysin gene.

Although a number of studies have investigated the interactions between GBS and phagocytes, only one employs flow cytometry to measure phagocytic engulfment and superoxide production[12]. In that study the authors report that phagocytosis of GBS and superoxide production after ingestion are greater for neutrophils than for monocytes, but it does not evaluate the effect of pregnancy, which may explain the differences from our study. In addition to differences in study populations, McCloskey and Salo use GBS incubated with antibody and complement to examine opsonophagocytosis. In contrast, our study used unopsonized GBS to examine engulfment.

In that colonization status, as well as pregnancy, defined our subject populations, we were aware that sample collection techniques would inevitably lead to variations in GBS recovery rates. We elected to allow the physician to determine the collection procedure. As only lower-vaginal segment GBS colonization was examined in our study, GBS carriage may have been modestly underestimated. However, statistical sample size evaluation suggested that the addition of more subjects was unlikely to alter the conclusions of the study.

The results of our investigations indicated a unique interaction between GBS and monocytes from pregnant, GBS-colonized women. GBS may accumulate in large numbers in monocytes from colonized, pregnant women, which tend to release their superoxide into the extracellular milieu. Superoxide is most likely to be more potent when sequestered in an intracellular location; therefore, these monocytes, which have released their superoxide, may in effect create an intracellular niche for GBS. Other recent studies support the idea that monocytes provide an intracellular haven for GBS. Several groups have reported that GBS can persist intracellularly in murine macrophages for 24–48 h following infection[13,14], as well as inactivate the protein kinase C pathway, one of the main signal transduction pathways leading to macrophage activation[14].

The alterations in innate immune response by monocytes from pregnant, colonized women demonstrated here provide the initial evidence that the immune system, which should function in a protective fashion, may not contribute to the prevention of GBS colonization. These alterations are by no means the only contributory factors in the colonization process, but provide a framework for further dissection of the underlying mechanism. In the process of elucidating the mechanism leading to GBS colonization, more information on the immune response to GBS will be generated, and may provide a clearer path to the design of an effective GBS vaccine.

### ACKNOWLEDGEMENTS

We thank Sandra White for her assistance in obtaining the blood samples from the pregnant participants, and Pamela Staton for her assistance in obtaining blood samples from the nonpregnant participants.

These studies were presented in part at the International Infectious Disease Society for Obstetrics and Gynecology – USA Fifth Annual Conference on Women's Health, held May 2000 in San Francisco, CA.

### REFERENCES

1. Boyer KM, Gotoff SP. Prevention of early-onset neonatal group B streptococcal disease with selective intrapartum chemoprophylaxis. *N Engl J Med* 1986;314:1665–9
2. Schuchat A. Epidemiology of group B streptococcal disease in the United States: shifting paradigms. *Clin Microbiol Rev* 1998;11:497–513
3. Arnaout MA. Structure and function of the leukocyte adhesion molecules CD11/CD18. *Blood* 1990;75:1037–50
4. Malech HL, Gallin JI. Current concepts: immunology. Neutrophils in human diseases. *N Engl J Med* 1987;317:687–94
5. Koumandakis E, Koumandakis I, Kalamani E, *et al.* Enhanced phagocytosis of mononuclear phagocytes in pregnancy. *Br J Obstet Gynaecol* 1986;93: 1150–4
6. Shibuya T, Izuchi K, Kuriowa A, *et al.* Study on nonspecific immunity in pregnant women: II. Effect of hormones on chemiluminescence response of peripheral blood phagocytes. *Am J Reprod Immunol* 1991;26:76–81
7. Barriga C, Rodriguez AB, Ortega E. Increased phagocytic activity of polymorphonuclear leukocytes during pregnancy. *Eur J Obstet Gynecol Reprod Biol* 1994;57:43–6
8. Winram SB, Jonas M, Chi E, Rubens CE. Characterization of group B streptococcal invasion of human chorion and amnion epithelial cells *in vitro*. *Infect Immun* 1998;66:4932–41
9. Gibson RL, Lee MK, Soderland C, *et al.* Group B streptococci invade endothelial cells: type III capsular polysaccharide attenuates invasion. *Infect Immun* 1993;61:478–85
10. Nizet V, Gibson RL, Chi EY, *et al.* Group B streptococcal beta-hemolysin expression is associated with injury of lung epithelial cells. *Infect Immun* 1996;64:3818–26
11. Fettucciari K, Rosati E, Scaringi L, *et al.* Group B *Streptococcus* induces apoptosis in macrophages. *J Immunol* 2000;165:3923–33
12. McCloskey PS, Salo RJ. Flow cytometric analysis of group B streptococci phagocytosis and oxidative burst in human neutrophils and monocytes. *FEMS Immunol Med Microbiol* 2000;27:59–65
13. Valenti-Weigand P, Benkel P, Rohde M, Chhatwal GS. Entry and intracellular survival of group B streptococci in J774 macrophages. *Infect Immun* 1996;64:2467–73
14. Cornacchione P, Scaringi L, Fettucciari K, *et al.* Group B streptococci persist inside macrophages. *Immunology* 1998;93:86–95

RECEIVED 2/21/01; ACCEPTED 5/25/01
